# Comparison between the cranial magnetic resonance imaging features of neuromyelitis optica spectrum disorder versus multiple sclerosis in Taiwanese patients

**DOI:** 10.1186/s12883-014-0218-8

**Published:** 2014-11-30

**Authors:** Ming-Feng Liao, Kuo-Hsuan Chang, Rong-Kuo Lyu, Chin-Chang Huang, Hong-Shiu Chang, Yih-Ru Wu, Chiung-Mei Chen, Chun-Che Chu, Hung-Chou Kuo, Long-Sun Ro

**Affiliations:** Department of Neurology, Chang Gung Memorial Hospital-Linkou Medical Center and Chang Gung University College of Medicine, Taipei, Taiwan; Department of Neurology, Chang Gung Memorial Hospital, 199, Tung Hwa North Road, Taipei, Taiwan

**Keywords:** Neuromyelitis optica, Multiple sclerosis, Anti-aquaporin 4 antibody, Magnetic resonance imaging

## Abstract

**Background:**

Neuromyelitis optica spectrum disorder (NMOSD) and multiple sclerosis (MS) are inflammatory diseases of the central nervous system with different pathogenesis, brain lesion patterns, and treatment strategies. However, it is still difficult to distinguish these two disease entities by neuroimaging studies. Herein, we attempt to differentiate NMOSD from MS by comparing brain lesion patterns on magnetic resonance imaging (MRI).

**Methods:**

The medical records and cranial MRI studies of patients with NMOSD diagnosed according to the 2006 Wingerchuk criteria and the presence of anti-aquaporin 4 (anti-AQP4) antibodies, and patients with MS diagnosed according to the Poser criteria, were retrospectively reviewed.

**Results:**

Twenty-five NMOSD and 29 MS patients were recruited. The NMOSD patients became wheelchair dependent earlier than MS patients (log rank test; *P* = 0.036). Linear ependymal (28% vs. 0%, *P* = 0.003) and punctate lesions (64% vs. 28%, *P* = 0.013) were more frequently seen in NMOSD patients. Ten NMOSD patients (40%) had brain lesions that did not meet the Matthews criteria (MS were separated from NMOSD by the presence of at least 1 lesion adjacent to the body of the lateral ventricle and in the inferior temporal lobe; or the presence of a subcortical U-fiber lesion or a Dawson finger-type lesion). The different image patterns of NMOSD didn’t correlate with the clinical prognosis. However, NMOSD patients with more (≧10) brain lesions at onset became wheelchair dependence earlier than those with fewer (<10) brain lesions (log rank test; *P* < 0.001).

**Conclusions:**

The diagnostic sensitivity of NMOSD criteria can be increased to 56% by combining the presence of linear ependymal lesions with unmet the Matthews criteria. The prognoses of NMOSD and MS are different. A specific imaging marker, the linear ependymal lesion, was present in some NMOSD patients. The diagnosis of NMOSD can be improved by following the evolution of this imaging feature when anti-AQP4 antibody test results are not available.

## Background

Neuromyelitis optica (NMO) is an inflammatory disease mainly characterized by optic neuritis (ON) and longitudinally extensive spinal cord lesions (LESCLs) [1,2]. It displayed relapsing and remitting disease course and central nervous system (CNS) inflammation which is similar to Asian or optico-spinal form multiple sclerosis (OSMS).

The discovery of NMO biomarker anti-aquaporin-4 (anti-AQP4) antibody clearly separates these two diseases into different entities [1-3]. Anti-AQP4 antibody plays an important role in the pathogenesis of NMO. It is reported in around 61% ~ 90% of patients with NMO and in only 0% ~ 9% of MS patients [4-7]. Typical LESCLs are rarely seen in the patients with multiple sclerosis (MS). This clear distinction suggests that NMO and MS could be two different CNS inflammatory disorders with respect to their immunopathogenesis [4,8].

Some patients with anti-AQP4 antibody had recurrent optic neuritis, recurrent myelitis, optic neuritis and myelitis associated with systemic autoimmune disease or the brain lesions, rather than the typical features of optic neuritis and LESCLs. Those patients are regarded as having NMO spectrum disorder (NMOSD) [8,9]. The reported frequency of brain involvement in NMO patients ranges from 5% to 89% [10-25]. The brain lesions of NMOSD also become temporally and spatially disseminated, as do those of MS. Around 5.6% ~ 42% of brain lesions in NMOSD fulfill the Barkhof magnetic resonance imaging (MRI) criteria [11,13,16,19,20,26]. Although Matthews et al. have proposed criteria to distinguish NMOSD from MS by identifying characteristic cerebral lesions for NMOSD in the hypothalamus and periaqueductal area [10-16,20,26-30], validation, especially in Asians, is still required [27,31,32]. Given that the pathogenesis and treatment of NMOSD and MS are different, the standard immunomodulation therapy for MS, such as interferon, may be ineffective to control relapses or disease progression of NMO [8,33-35]. Specific imaging markers are needed to distinguish NMOSD from MS particularly in the early stage. By comparing the cranial magnetic resonance imaging (MRI) characteristics of NMOSD with those of MS, we found linear ependymal and punctate lesions specifically occurring in patients with NMOSD, whereas corpus callosum lesions are frequently seen in patients with MS. This clear difference in imaging features will be helpful in distinguishing NMOSD from MS in clinical practice.

## Methods

### Study population

We retrospectively reviewed the medical records of all NMO or MS patients with one or more episodes of CNS inflammatory disease treated between January 2009 and January 2014 at Chang Gung Memorial Hospital-Linkou Medical Center, a tertiary referral medical center in the northern Taiwan. A CNS inflammatory episode was defined as the presence of patient-reported symptoms or objectively observed typical signs of an acute inflammatory event including optic neuritis and myelitis in the CNS, with duration of at least 24 hours [36]. Patients with optic neuritis is diagnosed as having typical clinical symptoms of blurred vision and visual-field defect with evidence of a relative afferent pupillary defect (by neurological examination or prolonged p100 wave of visual evoke potential study) [37], or by the diagnosis of ophthalmologists. At the time of the attack, all patients were tested for anti-AQP4 antibodies to confirm the diagnosis. The protocol of this study was approved by the Institutional Review Board of Chang Gung Memorial Hospital and University (License no. 101-3410B).

### Diagnosis of NMOSD and MS

The diagnoses of NMO and MS were according to the revised 2006 Wingerchuk criteria [2] and Poser criteria [38], respectively. The NMO patients who also had brain lesions were diagnosed as NMOSD [8]. All NMOSD patients had both optic neuritis and myelitis during the follow-up periods. The serum anti-AQP4 antibodies were detected using an enzyme-linked immunosorbent assay (ELISA) system according to the manufacturer’s instructions (RSR/Kronus, Cardiff, UK) [39], and a level greater than 5 U/mL was considered seropositive. There are reports of combined autoimmune disease (myasthenia gravis or Sjögren’s syndrome) with NMOSD [40-43]. To simplify the study population, patients with Sjögren’s syndrome, systemic lupus erythematosus, rheumatoid arthritis, vasculitis, myasthenia gravis or underlying malignancies were excluded in our study.

### Collection of clinical and imaging data

**Figure 1:**
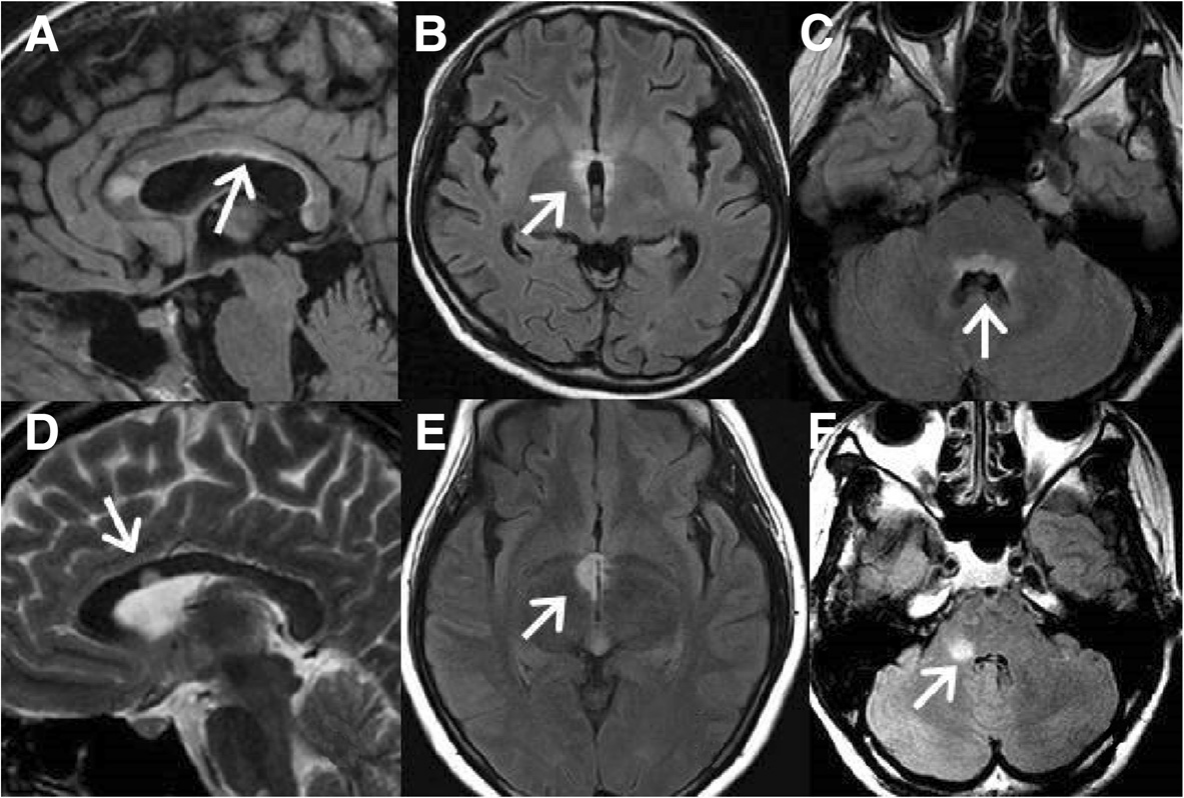
**Brain MRI findings of three NMOSD patients and three MS patients.** FLAIR MRI shows a **(A)** continuous linear ependymal lesion along the ventricle in a 29-year-old woman with NMOSD; **(B)** symmetrical hypothalamic lesion in an 18-year-old girl with NMOSD; and **(C)** typical symmetrical and continuous ependymal lesion around the periaqueductal area in a 17-year-old girl with NMOSD. T2-weighted image shows **(D)** ependymal dot lesions beside the ventricle in a 32-year-old woman with MS. FLAIR MRI shows **(E)** an asymmetric hypothalamic lesion in a 26-year-old woman with MS and **(F)** asymmetric ependymal dot lesions along the periaqueductal gray in a 37-year-old woman with MS. NMOSD: neuromyelitis optica spectrum disorder; MS: multiple sclerosis; FLAIR: fluid attenuated inversion recovery; MRI: magnetic resonance imaging. NMOSD: neuromyelitis optica spectrum disorder.

**Figure 2:**
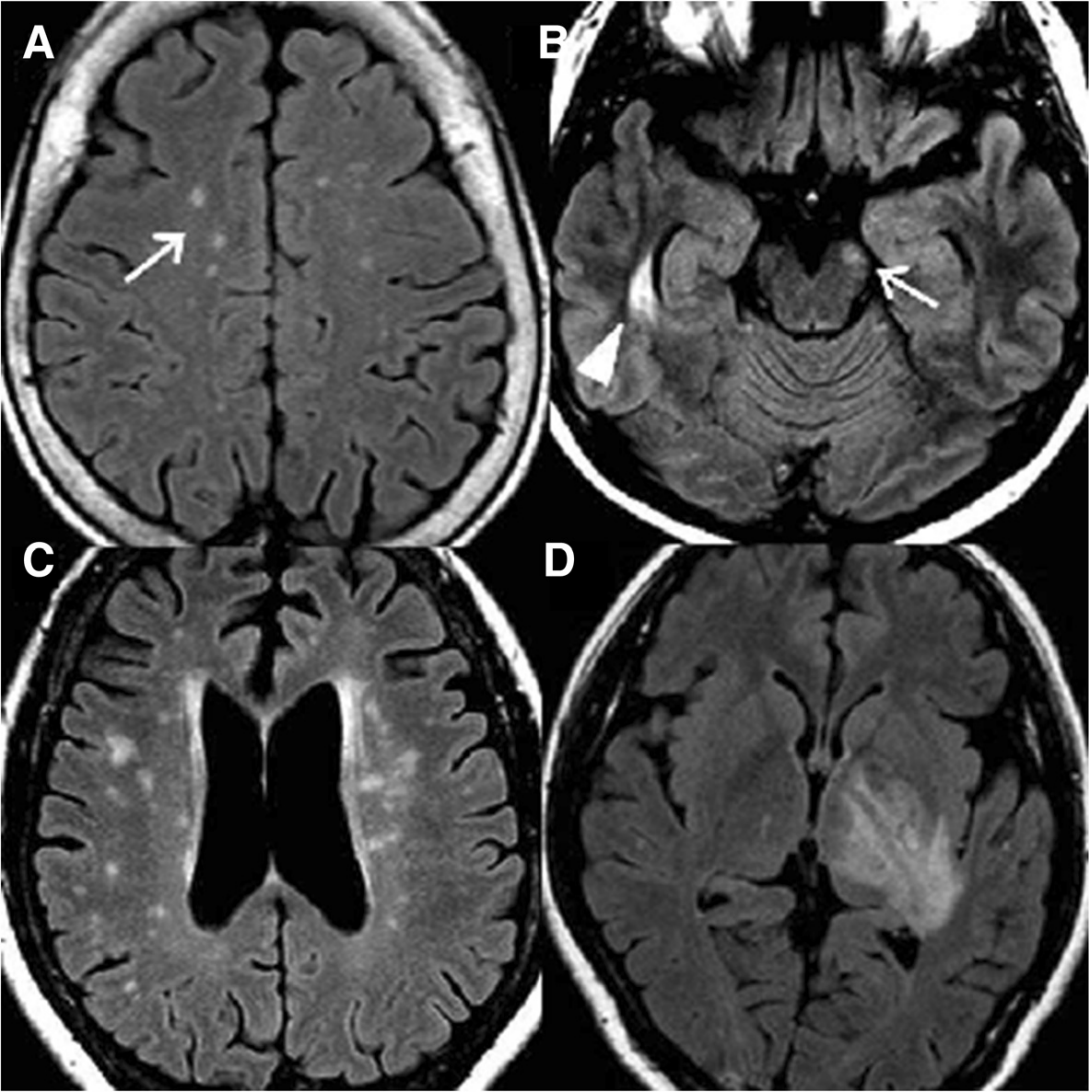
**Different brain magnetic resonance imaging characters of NMOSD.** Fluid attenuated inversion recovery (FLAIR) magnetic resonance imaging (MRI) shows a **(A)** punctate lesion (white arrow) on subcortical region in a 71-year-old woman with NMOSD; **(B)** right temporal (white arrow head) and left mid brain lesion (white arrow) in a 44-year-old girl with NMOSD; and **(C)** dawson finger like lesion around the bilateral subcortical and peri-ventricular region area in a 56-year-old girl with NMOSD; **(D)** tumefactive lesions on the left thalamus and posterior limb of internal capsule in a 34-year-old woman with NMOSD. NMOSD: neuromyelitis optica spectrum disorder.

**Figure 3:**
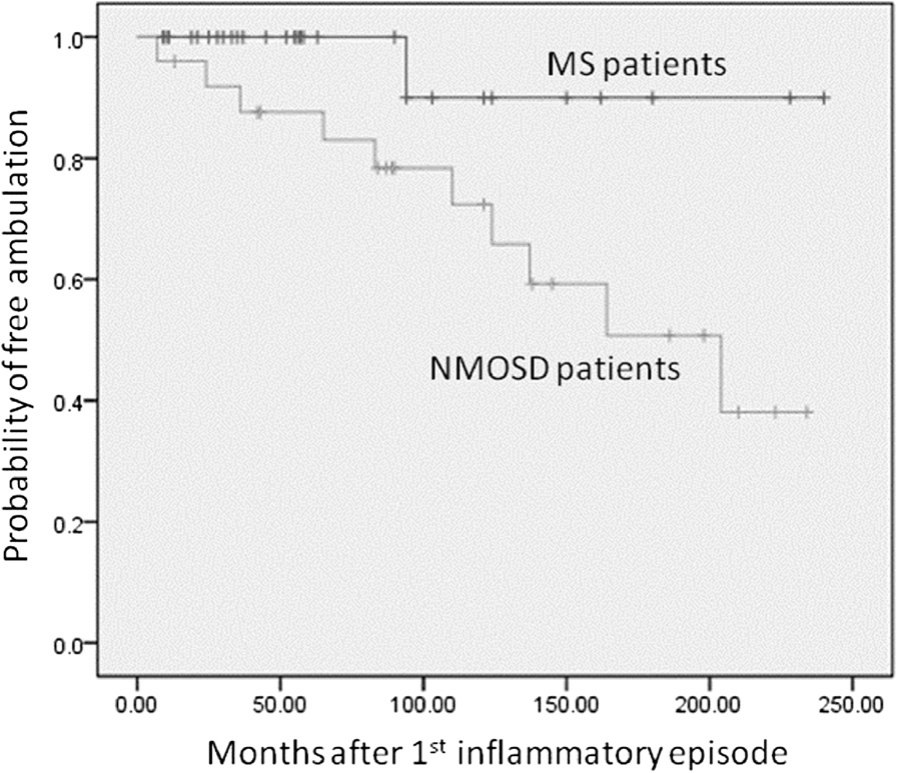
**Wheelchair dependence occurred earlier in NMOSD patients than MS patients (log rank test;** ***P*** **= 0.036).** NMOSD: neuromyelitis optica spectrum disorder; MS: multiple sclerosis.

We retrospectively reviewed patients’ clinical data including gender, age of onset, clinical symptoms, results of cerebrospinal fluid (CSF) analysis, and auto-antibody profiles (antinuclear antibody [ANA], rheumatoid factor [RF], and anti-Sjögren’s syndrome A [SSA]/ Sjögren syndrome B [SSB] antibodies). Cranial MRIs were taken during the clinical relapse; the protocols included T1 (repetition time [TR] = 250 ~ 760 ms, echo time [TE] = 1.8 ~ 20 ms), T1-enhanced, T2-weighted (TR = 2830 ~ 6400 ms, TE = 70 ~ 120 ms), and fluid-attenuated inversion recovery (FLAIR) sequencing images (TR = 7000 ~ 9800 ms, TE = 70 ~ 150 ms). The sections were obtained at 4 or 5 mm slice thickness. Several cranial MRI studies were carried out during the follow-up periods, and any abnormality in a series of MRI images was noted. The same MRI abnormalities detected by repeated MRI studies in the same patient were not counted again (no double counting). We used the term “linear ependymal lesions” (Figure 1A-C) to describe the symmetric and continuous sub-ependymal lesions extending in parallel to the ventricular surface including the periaqueductal gray matter surface, which is different from the ependymal irregularity known as the “dot-dash” sign previously described in MS patients [44,45]. The term “ependymal dot” indicated the asymmetric and non-continuous demyelinated lesions in the ependymal layer covering the periaqueductal and ventricular surface (Figure 1D-F). The MRI presentations of NMOSD patients were variable [14,27]. After reviewing brain images of those patients, we divided the MRI into three subtypes as follows: (1) linear ependymal type (Figure 1A-C); (2) punctate type (Figure 2A); (3) demyelination type (including dawson finger like lesions and large tumefactive demyelination lesions) (Figure 2B-D). All different image subtypes of MRI may show multiple brain lesions. We counted the brain lesions number (either small punctate lesions or large demyelination lesions) on the first MRI of NMOSD patients and evaluated its correlation with prognosis.

### Criteria for the evaluation of diagnostic accuracy

To distinguish MS from NMOSD on cranial MRIs, Matthews et al. proposed the presence of at least 1 lesion adjacent to the body of the lateral ventricle and in the inferior temporal lobe; or the presence of a subcortical U-fiber lesion or periventricular Dawson’s finger lesion [30]. The sensitivity, specificity, positive predictive value, and negative predictive value of using unmet the Matthews criteria and the presence of linear ependymal lesions to distinguish NMOSD from MS were tested in our patients.

### Statistical analysis

Statistical analyses were performed using Statistical Program for Social Sciences (SPSS) statistical software (version 13.0; Chicago, IL, USA). Fisher exact test was used to compare clinical symptoms and imaging features of NMOSD with those of MS. Non-categorical variables are expressed as the means ± standard deviation (SD) and compared by two-sample *t*-tests. Survival curves were estimated by the Kaplan–Meier method. Time zero for the survival analysis was taken as the date of the first CNS inflammatory episode. The primary end-point was the time when patients became wheelchair dependent (EDSS = 7). For patients who remained ambulatory, the follow-up period ended on the date of the last visit. All *P* values were two-tailed, and a *P* value less than 0.05 was considered statistically significant.

## Results

Of the 31 patients with NMO fulfilling the Wingerchuk 2006 criteria, 25 (81%) had brain lesions and were diagnosed as having NMOSD. All above patients had long-segment cervical or thoracic myelitis (≥3 segments) confirmed by MRI studies during follow-up periods. Of the 29 patients with MS meeting the Poser criteria, all fitted the clinical presentations of MS and had negative anti-AQP4 antibody tests. The age of onset in NMOSD patients (37.8 ± 13.6 years old) was similar to that in MS patients (33.7 ± 9.2 years old, Table 1). The follow-up period was longer in NMOSD patients (129.0 ± 69.5 months) than in MS patients (77.6 ± 64.2 months, *P* = 0.007). Sensory disturbances were the most common symptoms occurring during the follow-up period in both NMOSD patients (100%) and MS (79%) patients. Symptoms of weakness (100% vs. 69%, *P* = 0.002), sensory disturbance (100% vs. 79%, *P* = 0.025), blurred vision (100% vs. 66%, *P* = 0.001), and urine/stool retention (48% vs. 10%, *P* = 0.003) were more frequently seen in NMOSD patients. On the other hand, diplopia (34% vs. 8%, *P* = 0.025) and dysphagia/dysarthria (24% vs. 0%, *P* = 0.012) were more frequently seen in MS patients. Common endocrinopathies (diabetes mellitus, and thyroid dysfunction), polyuria (8% vs. 0%, *P* = 0.210), and hiccup (8% vs. 0%, *P* = 0.210) occurred with similar frequency in both groups. Five NMOSD patients but no MS patient had respiratory failure (20% vs. 0%, *P* = 0.017) and two of these five died from pneumonia and sepsis. Eleven of 25 (44%) NMOSD patients and 1 of 29 (3.4%) MS patients became wheelchair dependent during the follow-up periods (log rank test; *P* = 0.036) (Figure 3).

**Table 1:** **Demographic data of evaluable NMOSD and MS patients during the follow-up period**

	**NMOSD (n = 25)**	**MS (n = 29)**	***P*** **-value**
Age at 1^st^ attack (years old)	37.8 ± 13.6	33.7 ± 9.2	0.194
Male: female	3 : 22	7 : 22	0.310
Symptoms during the study period			
Weakness (%)	25(100)	20(69)	0.002*
Sensory disturbance (%)	25(100)	23(79)	0.025*
Blurred vision (%)	25(100)	19(66)	0.001*
Consciousness change (%)^†^	8(32)	1(3)	0.008*
Diplopia (%)	2(8)	10(34)	0.025*
Dysphagia/dysarthria (%)	0(0)	7(24)	0.012*
Urine/stool retention (%)	12(48)	3(10)	0.003*
Hiccup (%)	2(8)	0(0)	0.210
Polyuria >3000 ml/day (%)	2(8)	0(0)	0.210
Endocrinopathy (%)	7(28)	3(10)	0.160
Diabetes mellitus (%)	3(12)	2(7)	0.653
Thyroid dysfunction (%)	4(16)	1(3)	0.170
Respiratory failure (%)	5(20)	0(0)	0.017*
Expired (%)	2(8)	0(0)	0.210
Follow-up duration (months)	129.0 ± 69.5	77.6 ± 64.2	0.007*
Annual relapse rate (%)	65.0 ± 50.1	54.4 ± 47.3	0.427
AQP4 antibody (%)	25 (100)	0 (0)	0.000*
Long term steroid, IST and DMT			
Steroid	22 (88)	8 (28)	0.000*
Steroid + Azathioprine	5 (20)	0 (0)	0.017*
Interferon beta	2 (8)	10 (35)	0.025*
Copaxone	1 (4)	1 (3)	1.000
Fingolimod	0 (0)	9 (31)	0.002*

NMOSD: neuromyelitis optica spectrum disorder; MS: multiple sclerosis; IST: immunosuppressant therapy; DMT: disease modifying therapy.

*Statistically significant difference between NMOSD and MS.

^†^Conscious change due to sepsis, epilepsy, shock, or other brain structure lesions.

There were 40 brain MRI studies in 25 NMOSD patients and 54 brain MRI studies in 29 MS patients at relapses. Thirty (75%) relapsing episodes in NMOSD group received high dose (500 ~ 1000 mg methylprednisolone/day) intravenous steroid therapies, while 29 (54%) relapsing episodes in MS group received the same treatment. Ten (25%) NMOSD patients had optic neuritis at their first disease attack and five patients received pulse therapy. On the other hand, only five (17.2%) MS patients had optic neuritis as their first clinical attack and three of them received pulse therapy during attack. For the long-standing immunological treatment, twenty-two (88%) NMOSD patients received oral steroid, and five (20%) took oral steroid and azathioprine concurrently. Only eight (27.6%) MS patients received oral steroid treatment. Ten (34.5%) MS patients received interferon beta, one (3.4%) had copaxone, and nine (31%) took fingolimod. Some NMOSD patients were diagnosed as OSMS before testing anti-AQP4 antibody and three patients still received treatment with interferon beta and copaxone (Table 1).

**Figure 4:**
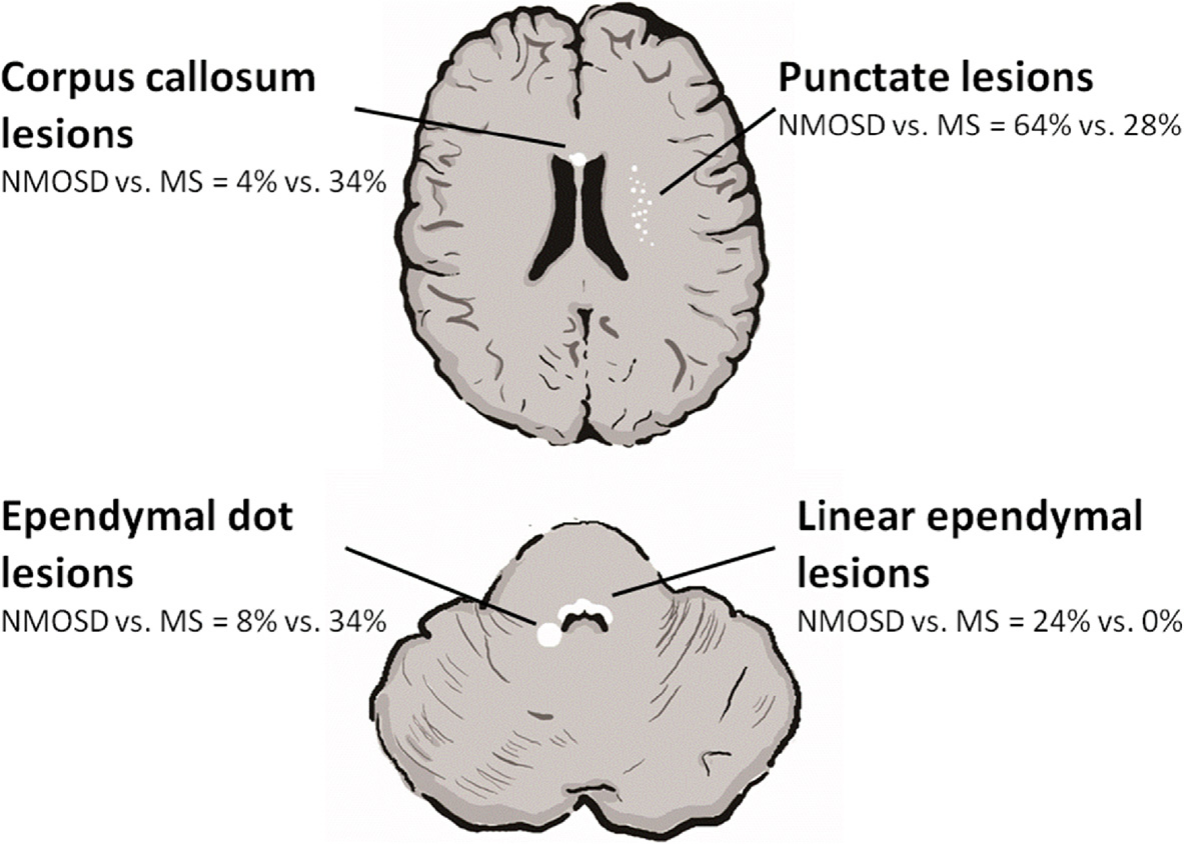
**Imaging characteristics of NMOSD and MS.** NMOSD: neuromyelitis optica spectrum disorder; MS: multiple sclerosis.

Linear ependymal lesions were present in 7 of 25 (28%, Figure 4) NMOSD patients but absent in MS patients (0%, *P* = 0.003, Table 2). Punctate lesions were more frequently seen in NMOSD than in MS patients (64% vs. 28%, *P* = 0.013). More MS patients (34%) demonstrated corpus callosum lesions than NMOSD patients (4%, *P* = 0.007). The spatial pattern of dissemination-in-space (DIS) pattern defined by the 2010 McDonald criteria [36] can be seen in 76% and 60% patients with MS and NMOSD, respectively (*P* = 0.449). The Mathews brain MRI pattern was seen in 79% of patients with MS and 60% of patients with NMOSD (*P* = 0.145). The frequencies of lesions located in the juxtacortical area, subcortical area, basal ganglion, periventricular area, temporal area, infratentorium, central/dorsal medulla, hypothalamus, and periaqueductal area (unilateral/bilateral) and the frequencies of juxtacortical U fibers, Dawson’s fingers, tumefactive, and ependymal dot lesions were similar on cranial MRIs of both groups.

**Table 2:** **Brain magnetic resonance imaging findings of evaluable NMOSD and MS patients**

	**NMOSD (n = 25)**	**MS (n = 29)**	***P*** **-value**
Number of MRI studies	40	54	
Location of lesions			
Juxtacortical region (%)	15(60)	20(69)	0.573
Subcortical region (%)	19(76)	21(73)	1.000
Basal ganglion (%)	15(60)	16(55)	0.787
Periventricular region (%)	15(60)	22(76)	0.250
Temporal lobe (%)	9(36)	13(45)	0.585
Infra-tentorium (%)	10(40)	20(69)	0.054
Central/dorsal medulla (%)	4(16)	7(24)	0.517
Corpus callosum (%)	1(4)	10(34)	0.007*
Periaqueductal gray(%)	8(32)	10(34)	1.000
Unilateral (%)	2(8)	8(28)	0.086
Bilateral (%)	6(24)	2(7)	0.125
Hypothalamus (%)	5(20)	2(7)	0.229
Fit McDonald criteria (DIS)	15(60)	22(76)	0.250
Fit Matthews criteria^†^	15(60)	23(79)	0.145
Morphological patterns			
U fiber lesion (%)	12(48)	18(62)	0.411
Dawson finger lesion (%)	11(44)	16(55)	0.586
Tumefactive lesion >3 cm (%)	2(8)	1(3)	0.591
Punctate lesion (%)	16(64)	8(28)	0.013*
Linear ependymal lesion (%)	7(28)	0(0)	0.003*
Ependymal dot lesion (%)	3(12)	10(34)	0.065

NMOSD: neuromyelitis optica spectrum disorder; MS: multiple sclerosis; McDonald criteria (DIS): 2010 McDonald dissemination in space criteria.

*Statistically significant difference between NMOSD and MS.

^†^The Matthews criteria used for separating MS from NMOSD: at least 1 lesion adjacent to the body of the lateral ventricle and in the inferior temporal lobe; or the presence of a subcortical U-fiber lesion or a Dawson finger-type lesion.

The specificity and sensitivity of using linear ependymal lesions to distinguish NMOSD from MS were high (100%) and low (24%), respectively (Table 3). The MRI findings in 10 NMOSD patients and 6 MS patients did not meet the Matthews criteria (sensitivity: 40%; specificity: 79%) for separating MS from NMOSD. The diagnostic sensitivity of these criteria for detecting NMOSD can be increased to 56% by combining the presence of linear ependymal lesions with unmet the Matthews criteria.

**Table 3:** **Diagnosis of NMOSD using different MRI criteria**

	**Sensitivity**	**Specificity**	**Positive predictive value**	**Negative predictive value**
1. Linear ependymal lesions	24% (6/25)	100% (29/29)	100%	60%
2. Unmet Matthews criteria*	40% (10/25)	79% (23/29)	63%	61%
3. 1 and 2	56% (14/25)	100% (29/29)	100%	73%

*The Matthews criteria used to separate MS from NMOSD: at least 1 lesion adjacent to the body of the lateral ventricle and in the inferior temporal lobe; or the presence of a subcortical U-fiber lesion or a Dawson’s finger-type lesion.

NMOSD: neuromyelitis optica spectrum disorder.

There were six (24%) and nineteen (76%) NMOSD patients had abnormal MRI studies at the first clinical attacks and during the follow-up periods, respectively. The annual relapse rate (0.614 ± 0.455 vs. 0.661 ± 0.526, *P* = 0.846) and the possibility of wheelchair dependent during the follow-up periods are similar between them (log rank test; *P* = 0.401).

We found that 6 (24%), 7 (28%), and 12 (48%) NMOSD patients demonstrated brain images fitting the characteristics of punctate lesion, linear ependymal lesion, and demyelination like lesion, respectively. The different image patterns did not significantly correlate with their clinical prognosis. The time to become wheelchair dependent were similar among those three groups (log rank test; *P* = 0.271). The annual relapse rate of linear ependymal group was possibly higher than demyelination group (0.93 ± 0.61 vs. 0.46 ± 0.40, *P* = 0.060).

**Figure 5:**
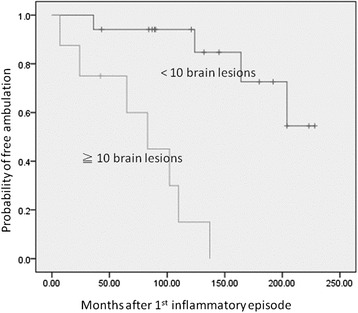
**NMOSD patients with more (≧10) brain lesions at onset became wheelchair dependence earlier than those with fewer (<10) brain lesions (log rank test;** ***P*** **< 0.001).** NMOSD: neuromyelitis optica spectrum disorder. ≧10 brain lesions: 10 or more brain lesions on patients’ first MRI studies. <10 brain lesions: <10 brain lesions on patients’ first MRI studies.

Eight (32%) NMOSD patients had 10 or more (≧10) brain lesions on their first MRI, These patients had higher wheelchair dependent rate than the NMOSD patients with fewer brain lesions (<10) during the follow-up periods (log rank test; *P* < 0.001). The annual relapse rate was possibly higher in the patients with more (≥10) than those with fewer (<10) brain lesions (0.92 ± 0.69 vs. 0.52 ± 0.34, *P* = 0.068) (Figure 5).

## Discussion

In our series, brain lesions became apparent in 81% of NMOSD patients during the follow-up periods. Symmetric linear ependymal lesions were an imaging feature in about one fourth (7/25) of NMOSD patients with brain lesions but in none of the MS patients. This MRI feature could therefore be used to distinguish NMOSD from MS with a high specificity (100%) but a low sensitivity (24%). We proposed that combining the presence of ependymal lesions with unmet the Matthews criteria would increase the sensitivity of the criteria to differentiate NMOSD from MS (56%), while maintaining their high specificity (100%), high positive predictive value (100%), and high negative predictive value (73%).

The frequency of brain involvement in NMO differs between Eastern and Western countries [10-25]. Brain lesions developed in more than 60% of NMO patients in studies from Japan [21,22] and Korea [13,14] and in less than 30% of NMO patients in studies from France [18], Italy [24], and the Caribbean [25]. Moreover, the distribution of brain lesions in NMOSD differs as well. In a study from the UK [30], few NMO patients had brains with Dawson’s finger-type lesions (0%), subcortical U-fiber lesions (0%), inferior temporal lobe lesions (19%), and periventricular lesions (23%), whereas Korean and Japanese NMOSD patients had brains with more periventricular lesions (40%) and ovoid shaped brain lesions (31.6%), respectively [14,16]. Similar to findings in other Asian countries, our results showed Dawson’s finger type (ovoid) lesions (44%), U-fiber shaped lesions (48%), inferior temporal lesions (36%), and periventricular lesions (60%) were frequently present in NMOSD [14,16]. Although Matthews et al. showed that periventricular, inferior temporal lobe, and U-fiber or Dawson’s finger-type lesions could be very sensitive (>90%) markers for distinguishing MS from NMOSD in UK populations, we showed that they were less sensitive in Taiwanese patients. However, by combining the presence of linear ependymal lesions with unmet the Matthews criteria, we could increase the sensitivity of these criteria from 40% to 56%. It is still uncertain whether genetic or environmental factors contribute to differences in brain lesion distribution.

The characteristic linear ependymal lesions of NMOSD in this study may be explained by the spatial pattern of AQP4, which is mainly expressed in astrocytes in the optic nerve, spinal cord, hypothalamus, and periependymal area in contact with CSF [34,46]. In a pathological study of rats, the AQP4 protein was abundant in glial cells bordering the subarachnoid space and ventricles [47], which is consistent with the distribution of brain lesions in our NMO patients. Moreover, in support of this speculation, Pittock et al. noted that the locations of NMO brain lesions are associated with structures expressing high levels of AQP4 [29]. Other studies also reported similar periependymal brain lesions in NMOSD patients [10,11,14,15,26,29]. Anti-AQP4 antibodies may penetrate the blood–brain barrier, bind to these AQP4-rich regions, and then initiate downstream immunopathological cascades responsible for the development of linear inflammatory lesions in the periependymal tissue of NMOSD patients. Thus, the identification of “linear ependymal lesions” on cranial MRI could be a useful imaging characteristic for identifying NMOSD.

The number of brain MRI lesions in the patients with clinically isolated syndromes (CIS) may predict the development of MS and correlate with disability status after 20 years [48]. Our study also shows the number of brain lesions at onset is an important prognostic factor for NMOSD. NMOSD patients that had 10 or more (≧10) brain lesions on their first MRI may be bound to wheelchairs more rapidly than those with fewer (<10) brain lesions, indicating the application of high potency immunosuppression on this group of patients.

In the present study, a few clinical features were found to distinguish between these two diseases. NMOSD patients with brain lesions more frequently had symptoms of optic neuritis and severe myelitis including blurred vision, sensory disturbance, weakness, and urine/stool retention, while MS patients more frequently had diplopia and dysphagia/dysarthria. Hiccup is reported to be a unique clinical presentation of NMO in patients with periaqueductal lesions [49]. Both of our two patients with hiccup also had linear ependymal lesions in the periaqueductal region. One study also shows that endocrinopathies like amenorrhea, diabetes insipidus, and hypothyroidism develop in patients with recurrent optic neuromyelitis [50]. In this study, hiccup and polyuria (>3000 ml/d) were only seen in NMOSD patients, but because the patient number was too small, this difference between NMOSD patients and MS patients was not statistically significant. The natural history of untreated NMO is significantly worse than that of MS [8,33]. The 5-year survival rate of NMO patients was only 68% in an earlier study [51]. Similarly in our study, five NMOSD patients had respiratory failure during the follow-up periods and two of them died from pneumonia and sepsis around 8 years and 23 years after the first symptoms onset, whereas none of our MS patients had these serious complications. The greater likelihood of wheelchair dependence in NMOSD patients is probably due to longitudinally extensive and greater spinal cord involvement.

This retrospective study may suffer from recruitment bias as a result of the small study population, and the treatment and follow-up durations were inconsistent in both groups. Nevertheless, in our study, prognosis was poorer in NMOSD patients than MS patients. A characteristic imaging marker, linear ependymal lesions, was only present in NMOSD patients but not MS patients. Thus, the differentiation of NMOSD from MS can be improved by closely monitoring the evolution of this image marker.

## Conclusions

In summary, NMOSD patients became wheelchair dependence earlier than MS patients. Furthermore, NMOSD patients who had more (≧10) brain lesions on their first MRI had a worse prognosis than those with fewer (<10) brain lesions. A specific imaging marker, the linear ependymal lesion, was present in some NMOSD patients. The diagnostic sensitivity of NMOSD criteria can be increased by combining the presence of linear ependymal lesions with unmet the Matthews criteria.

### Consent

The Institutional Review Boards of the Chang Gung Memorial Hospital waived the need for individual informed consent because all data were anonymized and de-identified prior to analysis.
